# Pseudo-honeycombing in a patient with coronavirus disease 2019 pneumonia

**DOI:** 10.1590/0037-8682-0656-2021

**Published:** 2022-02-25

**Authors:** Bruno Hochhegger, Gláucia Zanetti, Edson Marchiori

**Affiliations:** 1 Universidade Federal de Ciências da Saúde de Porto Alegre, Porto Alegre, RS, Brasil.; 2 Universidade Federal do Rio de Janeiro, Rio de Janeiro, RJ, Brasil.

**FIGURE 1:**
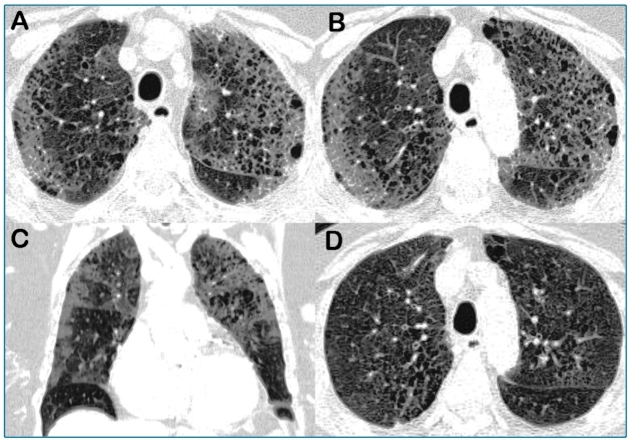
Chest computed tomography (CT) images with axial (A, B) and (C) coronal reconstruction shows small cystic formations sharing walls and ground-glass opacities predominating in the upper lobes. This pattern was suggestive of honeycombing. (D) A chest CT examination performed one year previously demonstrates centrilobular emphysema in the upper lobes.

An 85-year-old woman with chronic obstructive pulmonary disease and a smoking history of 40 packs per year was admitted to the emergency room with a 9-day history of dyspnea, fever, cough, and diffuse myalgia. She tested positive for coronavirus disease 2019 (Covid-19). A chest computed tomography (CT) examination performed eight days after symptom onset demonstrated bilateral ground-glass opacities associated with a pattern suggestive of honeycombing cysts (fibrosis), predominantly in the upper lobes (Figure 1A-C). A chest CT examination performed one year previously demonstrated centrilobular emphysema in the upper lobes (Figure 1D). Thus, the diagnosis of ground-glass opacities secondary to Covid-19 pneumonia associated with centrilobular emphysema was made. The patient recovered uneventfully with disappearance of symptoms and normalization of laboratory parameters. The patient was discharged after 14 days in an asymptomatic state.

Recent studies have documented the development of fibrosis, both histologically and radiologically, during the evolution of Covid-19 pneumonia^1^ ^,^ ^2^. Although pulmonary fibrosis occurs late (after Covid-19) in most cases, some reports show that it can appear in the early stages of the disease^3^. Our patient had a tomographic pattern suggestive of fibrosis with honeycombing, although the onset of the clinical picture had occurred only about one week previously. The superimposition of ground-glass opacities over the centrilobular emphysema detected one year previously in this patient simulated the honeycomb pattern, leading to the diagnosis of pseudo-honeycombing. Assistant physicians must be aware of possible previous lung parenchyma alterations and confounding factors for imaging diagnoses.
